# Three-Dimensional Digital Capture of Head Size in Neonates – A Method Evaluation

**DOI:** 10.1371/journal.pone.0061274

**Published:** 2013-04-08

**Authors:** Sascha Ifflaender, Mario Rüdiger, Arite Koch, Wolfram Burkhardt

**Affiliations:** Department of Neonatology and Paediatric Intensive Care, University Hospital Carl Gustav Carus, Dresden, Germany; Hôpital Robert Debré, France

## Abstract

**Introduction:**

The quality of neonatal care is mainly determined by long-term neurodevelopmental outcome. The neurodevelopment of preterm infants is related to postnatal head growth and depends on medical interventions such as nutritional support. Head circumference (HC) is currently used as a two-dimensional measure of head growth. Since head deformities are frequently found in preterm infants, HC may not always adequately reflect head growth. Laser aided head shape digitizers offer semiautomatic acquisition of HC and cranial volume (CrV) and could thus be useful in describing head size more precisely.

**Aims:**

1) To evaluate reproducibility of a 3D digital capture system in newborns. 2) To compare manual and digital HC measurements in a neonatal cohort. 3) To determine correlation of HC and CrV and predictive value of HC.

**Methods:**

Within a twelve-month period data of head scans with a laser shape digitizer were analysed. Repeated measures were used for method evaluation. Manually and digitally acquired HC was compared. Regression analysis of HC and CrV was performed.

**Results:**

Interobserver reliability was excellent for HC (bias-0.005%, 95% Limits of Agreement (LoA) −0.39–0.39%) and CrV (bias1.5%, 95%LoA-0.8–3.6%). Method comparison data was acquired from 282 infants. It revealed interchangeability of the methods (bias-0.45%; 95%LoA-4.55–3.65%) and no significant systematic or proportional differences. HC and CrV correlated (r^2^ = 0.859, p<0.001), performance of HC predicting CrV was poor (RSD ±24 ml). Correlation was worse in infants with lower postmenstrual age (r^2^ = 0.745) compared to older infants (r^2^ = 0.843).

**Discussion:**

The current practice of measuring HC for describing head growth in preterm infants could be misleading since it does not represent a 3D approach. CrV can vary substantially in infants of equal HC. The 3D laser scanner represents a new and promising method to provide reproducible data of CrV and HC. Since it does not provide data on cerebral structures, additional imaging is required.

## Introduction

The quality of neonatal care is mainly determined by long-term neurodevelopmental outcome. As survival rates for extremely preterm infants have improved, the number of preterm infants with poor neurodevelopmental outcome has not fallen over the past decade [1].

The neurodevelopment is affected not only by cerebral lesions such as hemorrhage or leucomalacia but also by brain growth. Reduced total brain volumes in preterm infants have been shown to correlate with poorer cognitive performance at school age [2]. In infants without structural lesions, head growth is mainly determined by severity of neonatal illness and medical interventions, such as nutrition during the initial hospital stay [3]–[6]. To optimize neonatal care with respect to head growth, an accurate and reliable longitudinal monitoring of head size is required.

Whereas cranial ultrasound and cerebral MRI are mandatory to detect and monitor structural lesions of the brain [7], repeated manual measurements of head circumference (HC) are routinely used to monitor head growth in infants without brain lesions. HC measurements in term and preterm infants correlate with neurodevelopment, but are not always reliable in the clinical context [8]. Both, circumference measurements below the 10^th^ percentile at 18–22 months of corrected age and velocity of changes in HC (head growth) during initial hospital stay are significantly linked to worse neurodevelopmental outcomes [9]–[11].

Frontal-occipital head circumference is well-correlated with total brain volume in the term newborn and in preterm infants at term with normal head shapes [12], [13]. However, postnatal moulding and subsequent head deformities such as dolichocephaly or positional plagiocephaly have been previously described in small preterm infants in the early postnatal period [14], [15] and have been associated with lower developmental scores at preschool age [16]. In these infants, two-dimensional measurements of head circumference may not always adequately reflect cranial volume and infants with similar HC will have considerable differences of total brain volumes.

Noninvasive laser shape digitizers capture a three-dimensional (3D) shape of infant's head. They are widely used in craniofacial surgery and fabrication of cranial remoulding orthoses in the therapy of deformational plagiocephalus [17]. These devices offer a semiautomatic acquisition of head circumference and other anthropometric measurements, such as cranial volume and cranial symmetry. It could be assumed that 3D digital capture of head shape could be a useful supplementary tool to monitor head growth in newborns.

Since there is no data available using laser shape digitizers to measure cranial volume (CrV) and HC in newborns, the present study was performed to test the following hypotheses: Infant head shape capturing with a 3D digital capture system is 1) a reproducible method, 2) an interchangeable method compared to manual HC measurements and 3) though there is a correlation of CrV and HC, CrV might vary substantially in infants with similar HC.

## Materials and Methods

### 3D digital capture device

A non-invasive laser shape digitizer (STARScanner, Orthomerica, Orlando, FL, USA) was used to acquire digital HC and volume data. The device captures a three-dimensional infant head shape using four Class-I eye-safe lasers that create circumferential light beams around the surface of the cranium. Eight cameras reconstruct the surface. The number of cameras and lasers are redundant to provide infant cranial shape acquisition in less than 2 seconds, thus reducing the need to restrain the motion of an active baby for a longer period of time. The device has been used clinically since 2001 to assist in the fabrication of cranial remoulding orthoses. It has received clearance from the FDA and is conform to European Standards and technical specifications for this use. The device is in use in over 100 centres in North America, South America, Europe, and Asia. It has previously been tested for accuracy and reliability in an infant head model and produced consistent measurements with inter-operator differences of less than 1 mm [18].

### Preparation of the infant

Before the infant is scanned, the infant's head is covered by stockinet to compress and mask the hair while allowing exposure of the face and both ears. Otherwise no other preparation is required. For scanning the infant has to be placed in the scanner for about 20 seconds. The scanning process lasts about 3 seconds, a period were the infant should not move. For hygienic purposes, surface of the scanner is always disinfected between scans.

### Digital measurements

**Figure 1:**
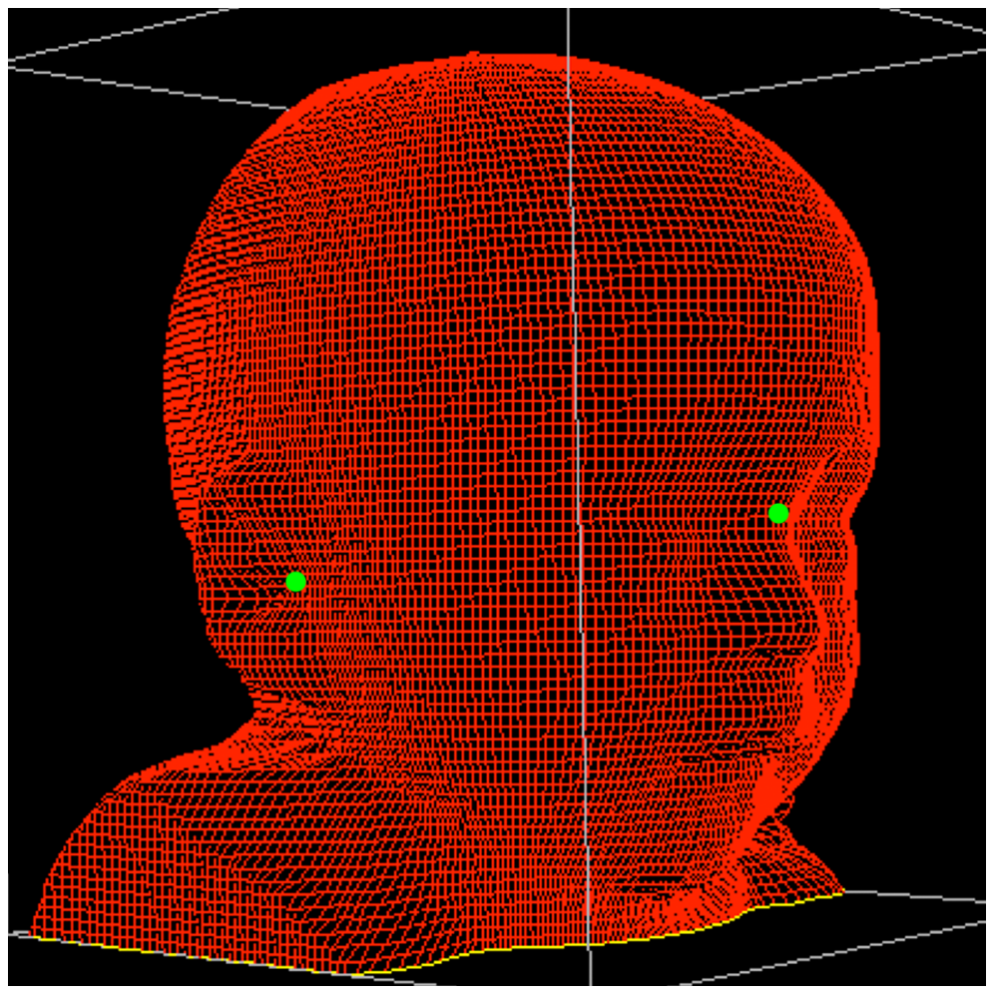
Definition of an anatomical reference plane. After incorporating scans into specialized software (YETI™ Shape Builder, Vorum Research Corporation, Vancouver, Canada) sellion and each tragion of the child are identified on the reconstructed image to define an anatomical reference plane. Green dots indicate the marked reference points.

Scans are incorporated into specialized software (YETI™ Shape Builder, Vorum Research Corporation, Vancouver, Canada). To define reproducible anatomical landmarks for subsequent calculations, a medical practitioner is setting markers at the sellion and each tragion on the reconstructed image (figure 1).

**Figure 2:**
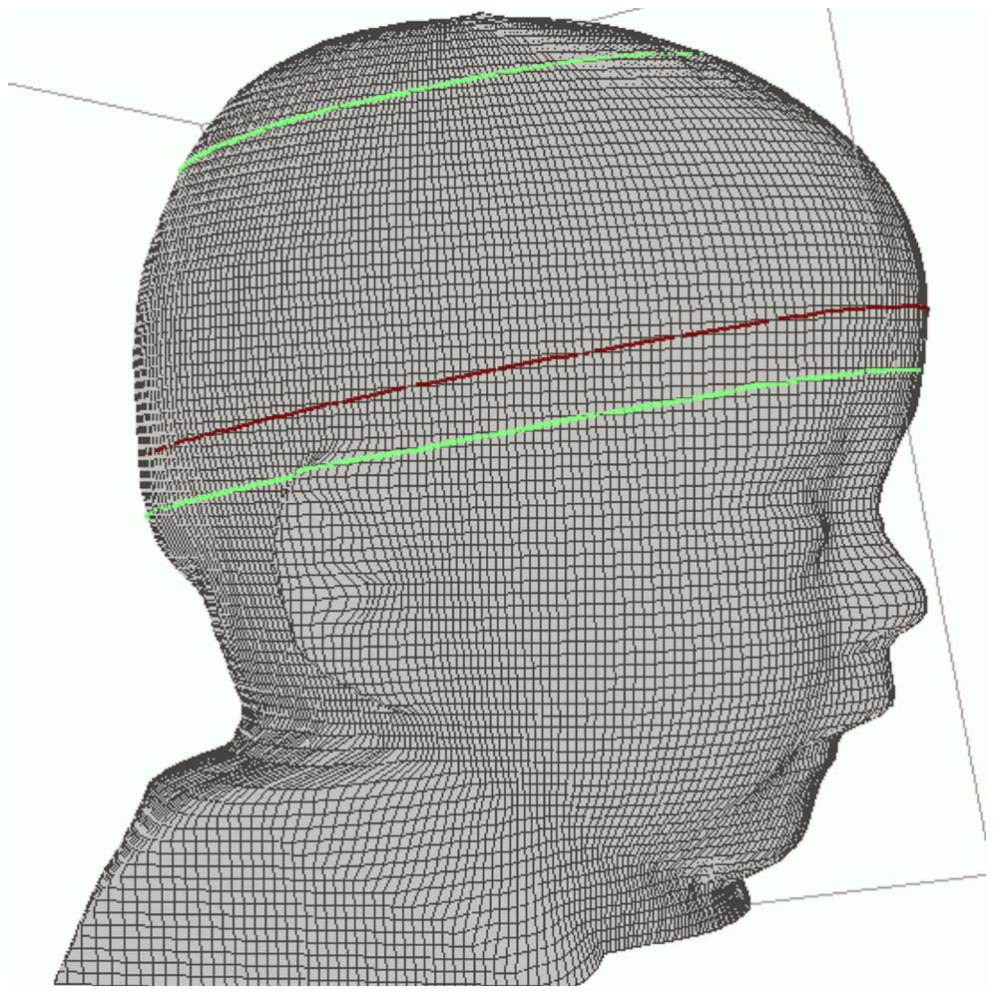
Measurement of head circumference and cranial volume. Measurement software (Cranial Comparison Utility, Vorum Research Corporation, Vancouver, Canada) is used to measure HC and CrV. Cranium is divided into 12 cross-sections parallel to the reference plane. Green lines indicate cross sections 2 and 8, used to measure CrV. Red line indicates cross section 3, used to measure HC.

By using another software (Cranial Comparison Utility, Vorum Research Corporation, Vancouver, Canada) the cranium is divided into twelve proportionally spaced cross-sections parallel to the reference plane. Cross sections 2–8 are used to calculate the cranial volume since these levels represent the skull shape without the soft tissue structures of the ears and face. Cross section 3 is used to measure head circumference, since it closely reflects the level of maximum frontal-occipital extension (figure 2).

### Data acquisition

Manual HC (HC_man_.) measurements and 3D head shape capture are performed weekly as part of the clinical routine at the intermediate care (IMC) of a tertiary neonatal unit in Dresden, Germany. HC_man_. is measured by attending nurses with a cloth tape. 3D head shape capture is routinely performed by a trained medical assistant once a week. All infants were included that were present on the IMC-unit at the time of measurement and had no peripheral IV at the scalp or required supplemental oxygen.

For the present study, we analyzed the first available scan of each infant and the values of corresponding manual HC measurements for the time span between April 2011 and March 2012. A trained medical assistant sets the landmarks and digitally acquired head circumference (HC_dig_.) and CrV were calculated automatically. Descriptive data were obtained from patient records including weight and length at birth, gestational age (GA) as well as postmenstrual age (PMA), weight, length and HC_man_. at the time of measurement.

### Intraobserver/Interobserver variability

Since technical accuracy and reliability of the device has been tested previously [18] it was the aim of the present study to test the reproducibility of data calculation depending on the setting of landmarks. Scan data of two randomly chosen patients were evaluated by five different trained and untrained observers to obtain intraobserver variability. Landmarks were set at sellion and both tragions by each observer on each object 10 times on different days, and data on HC and CrV was calculated by the software. Coefficients of variation (CV) were computed to assess intraobserver variability.

Scan data of 10 different patients were evaluated by two different trained observers to obtain interobserver data. Landmarks were set as described before and HC and CrV were calculated. Repeated measures were statistically evaluated for interobserver variability using Passing-Bablok-Regression [19] and Bland-Altman-Analysis [20].

### Statistical methods

Data were analysed and charted using GraphPad Prism version 5.0 for Windows/Mac OS (GraphPad Software, San Diego, CA, USA) and Microsoft Excel 2011 (Microsoft Corp., Redmond, WA, USA). Method comparison was performed using Passing-Bablok-Regression (PBR). PBR is a linear regression procedure with no special assumptions regarding the distribution of the samples and the measurement errors. The result does not depend on the assignment of the two methods [19]. The residual standard deviation (RSD) was calculated as a measure of the random differences between the two methods. Cusum test for linearity was used to evaluate how well a linear model fits the data. Passing-Bablok procedure was supplemented using Bland-Altman-Plot [20]. Bias and 95% limits of agreement were calculated and plotted. According to the recommendation of Dewitte et al [21], relative difference was used rather than absolute difference. Concerning interobserver reliability of manual HC measurements in previous investigations [8], limits of agreement within a 5% range were defined not to be clinically important for testing interchangeable use of the two methods. Mountain-Plot [22] was used as a complementary plot to the Bland-Altman-Analysis. It provides additional information about the distribution of the differences between the two methods. The relationship between HC and CrV was analysed using a linear regression model. The 95% prediction interval was used to show the variation of the data.

### Ethics statement

Ethical approval was given by the ethics committee of the Medical Faculty Carl Gustav Carus of the Technical University Dresden, Germany. As infants received standard care and routine measurements with approved devices, data collection was considered an audit of normal care. As only anonymized data was collected, ethics committee waived the need for specific parental consent.

## Results

Data was obtained from 282 infants during the one-year period. The clinical characteristics of the infants at birth and at the time of measurement are summarized in table 1.

**Table 1:** Clinical characteristics.

Data obtained at		Min.	25%	Median	75%	Max.
**birth**	GA [wks]	23+5	32+0	**34+2**	36+0	42+1
	weight [g]	545	1608	**2105**	2468	4230
	length [cm]	28.0	41.0	**44.0**	48.0	57.0
	HC [mm]	205	291	**310**	330	395
**measurement**	PMA [wks]	31+2	34+6	**35+6**	37+6	46+6
	PNA [d]	0	7	**12**	22	140
	weight [g]	1310	2001	**2225**	2604	4950
	length [cm]	38.0	44.0	**46.0**	49.0	57.0
	HC_man_ [mm]	270	305	**315**	330	388
	HC_dig_ [mm]	273.5	306.9	**319.2**	332.0	387.9
	CrV [ml]	208.2	289.2	**332.5**	368.6	630.6

Clinical characteristics at birth and at the time of head scan. GA = gestational age, HC = head circumference,PMA = postmenstrual age, PNA = postnatal age, HCman. = manually measured HC, HCdig. = digitally measured HC, CrV = cranial volume.

### Inter- and Intra-observer variability

**Figure 3:**
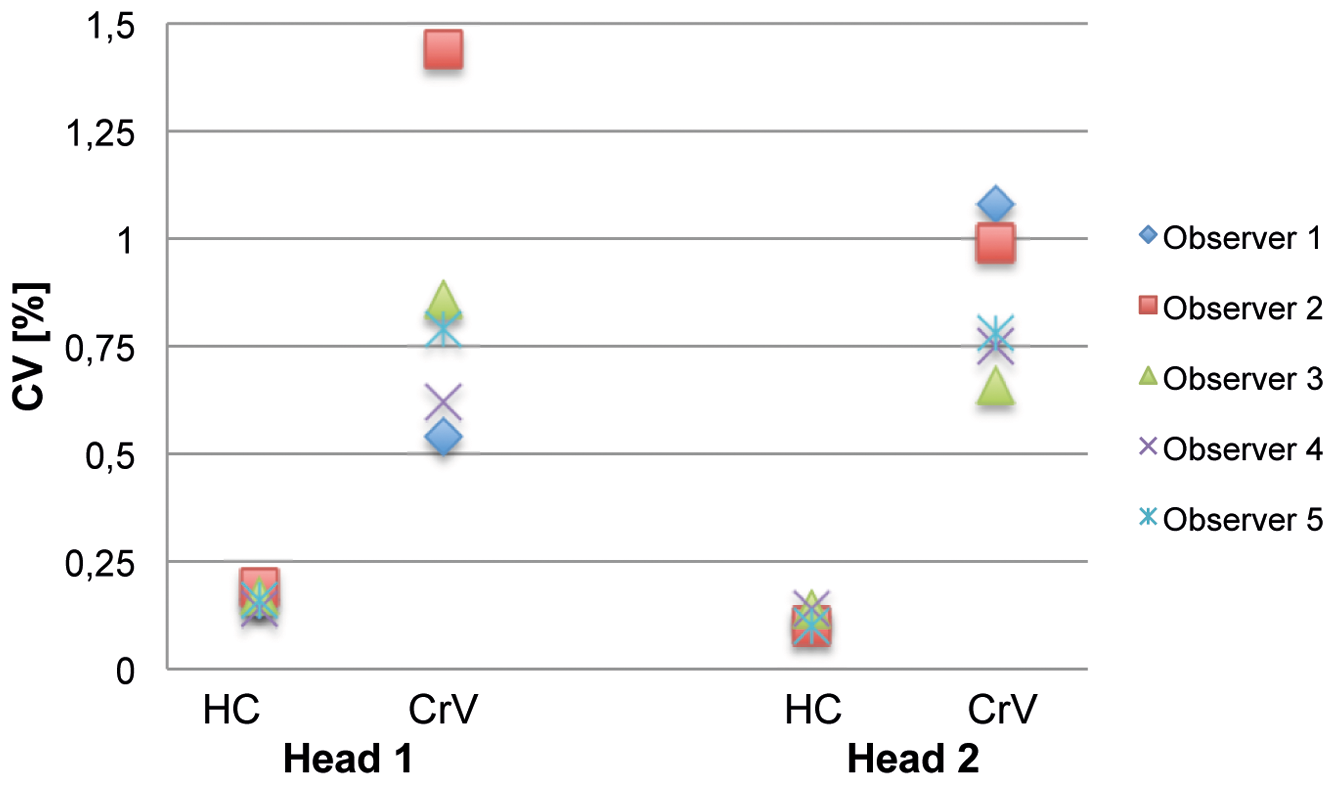
Intraobserver Variability of HC and CrV measurements. Coefficients of Variation (CV) of different observers (indicated by shape and colour of data points) are shown for HC and CrV of two different examined heads.

2 different heads were respectively measured 10 times by 5 different practitioners. Intraobserver Coefficients of Variation (CV) were calculated to test whether reliable multiple measures could be made by one observer (figure 3). Maximum CV was 0.2% for HC and 1.4% for CrV. Although there was a distinct difference in HC between the examined heads, there were no significant differences in CV.

For testing interobserver reliability ten randomly chosen head scans were evaluated by two different observers. Bland-Altman analysis showed good agreement between observers. For HC measurements differences were exiguous. Bias was −0.1±0.74 mm(−0.005±0.2%) with 95% Limits of Agreement (LoA) of −1.55 mm to 1.35 mm (−0.39 to 0.39%) and a standard deviation of residuals (RSD) of ±0.5 mm. Agreement of CrV measurements was good (bias 8.1±6.5 ml (1.5±1.1%), 95% LoA −4.6–20.8 ml (−0.8–3.6%), RSD ±5.8 ml).

### Comparison of manual and digital HC measurements

**Figure 4:**
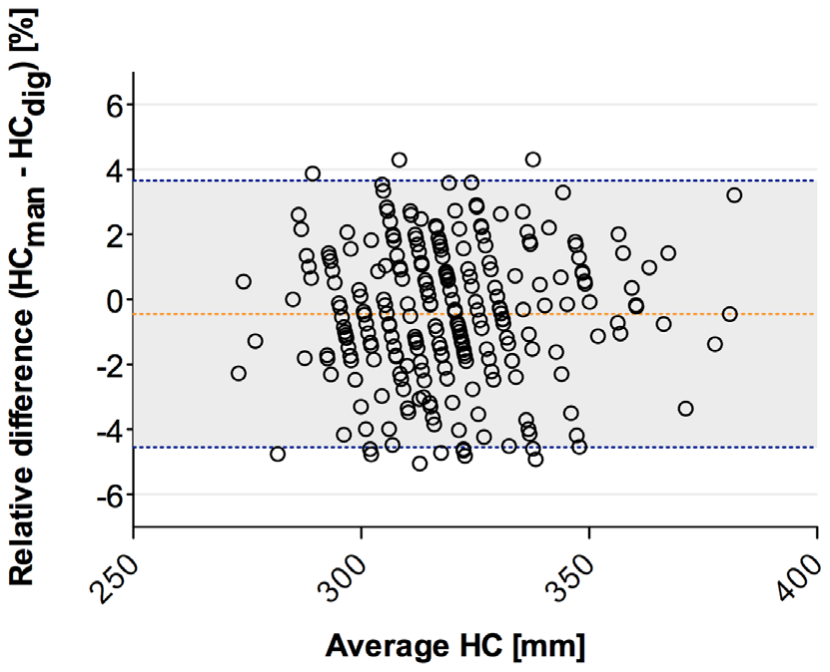
Comparison of manual and digital HC measurements. Bland-Altman-Plot showing the relative differences plotted over the means of both methods. Indicated are Bias (orange dashed line) and 95% Limits of Agreement (blue dashed lines).

**Figure 5:**
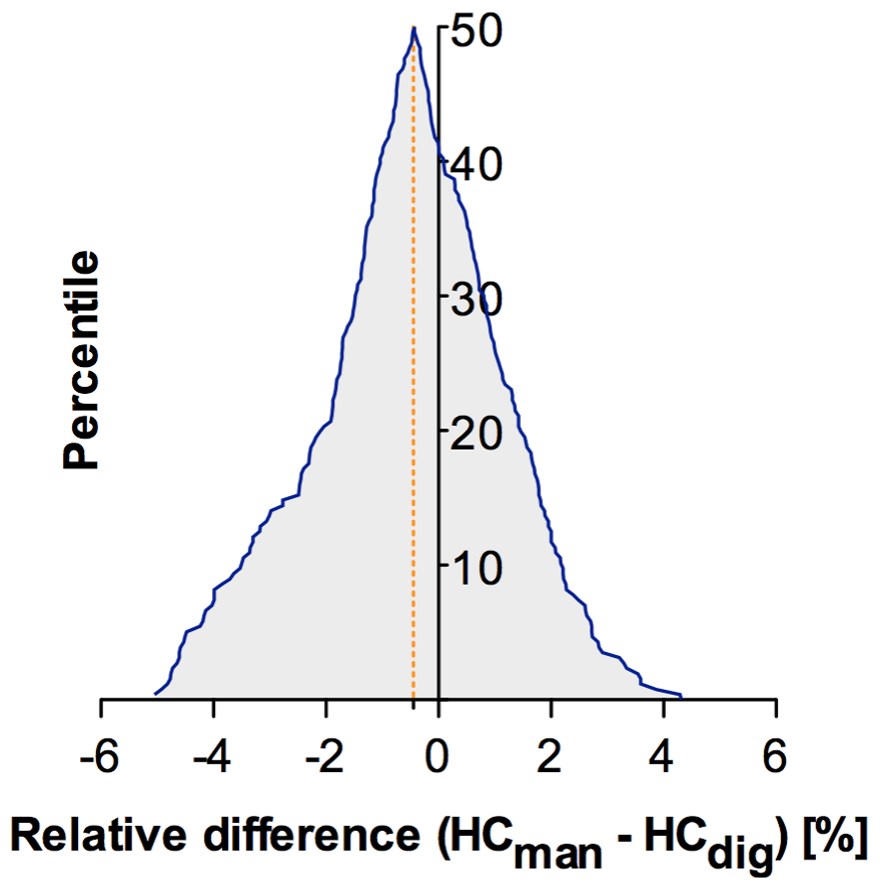
Distribution of differences between manual and digital HC measurements. Folded empirical cumulative distribution plot (Mountain-Plot). Percentiles of each ranked difference between the methods are plotted against the relative differences. The orange dashed line indicates bias.

Scans of 282 infants were analysed to compare manual and digital HC measurements. Bland-Altman revealed interchangeability of the two methods (bias −0.45±2.09%; 95%LoA −4,55–3.65%) as shown in figure 4. PBR showed no significant systematic or proportional differences (a = 1.001, 95%CI 0.96–1.05; ß = 1.17 95%CI −14.1–15.60). There was no significant deviation from linearity in cusum test (p = 0.63). Distribution of the differences between the two methods, shown in the Mountain-Plot in figure 5, reveals a mean difference close to zero with slightly lower values for manual measurements.

### Correlation and predictive value of head circumference and cranial volume

**Figure 6:**
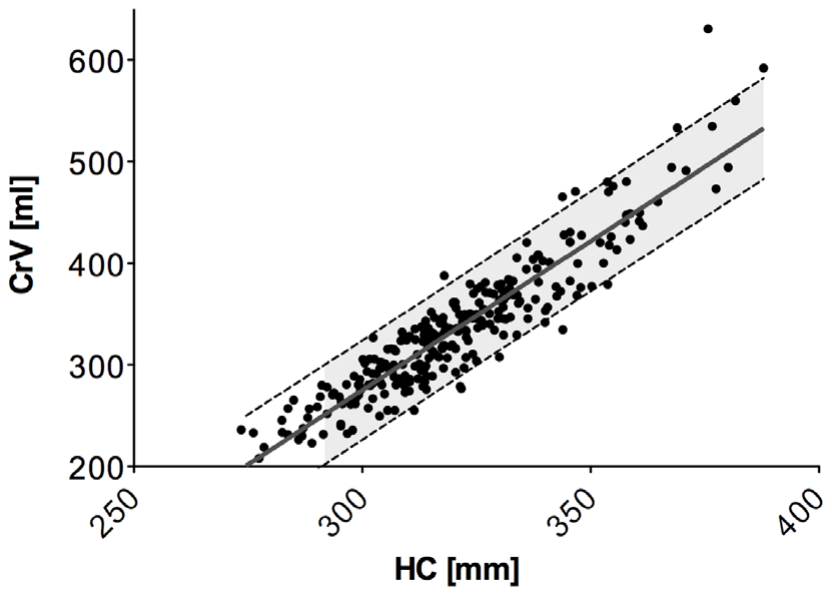
Correlation and prediction intervals of HC and CrV. Data points indicate CrV plotted against HC. Grey continuous line is indicating regression line. Dashed lines and light grey filling define the 95% prediction interval.

Despite of an overall correlation of HC and CrV (r^2^ = 0.859, p<0.001), regression analyses revealed a relatively poor performance in predicting CrV in individual patients from the correspondent HC. The residual standard deviation (RSD) of ±24 ml for predicting CrV seems to be of clinical relevance in a cohort of preterm infants with a median total CrV of 330 ml. As shown in figure 6, the 95% prediction interval illustrates a broad range of possible CrV values for equal HC values. As an example, in infants with a HC of 300 mm mean CrV was 275 ml, however the 95% prediction interval was ±49 ml.

**Figure 7:**
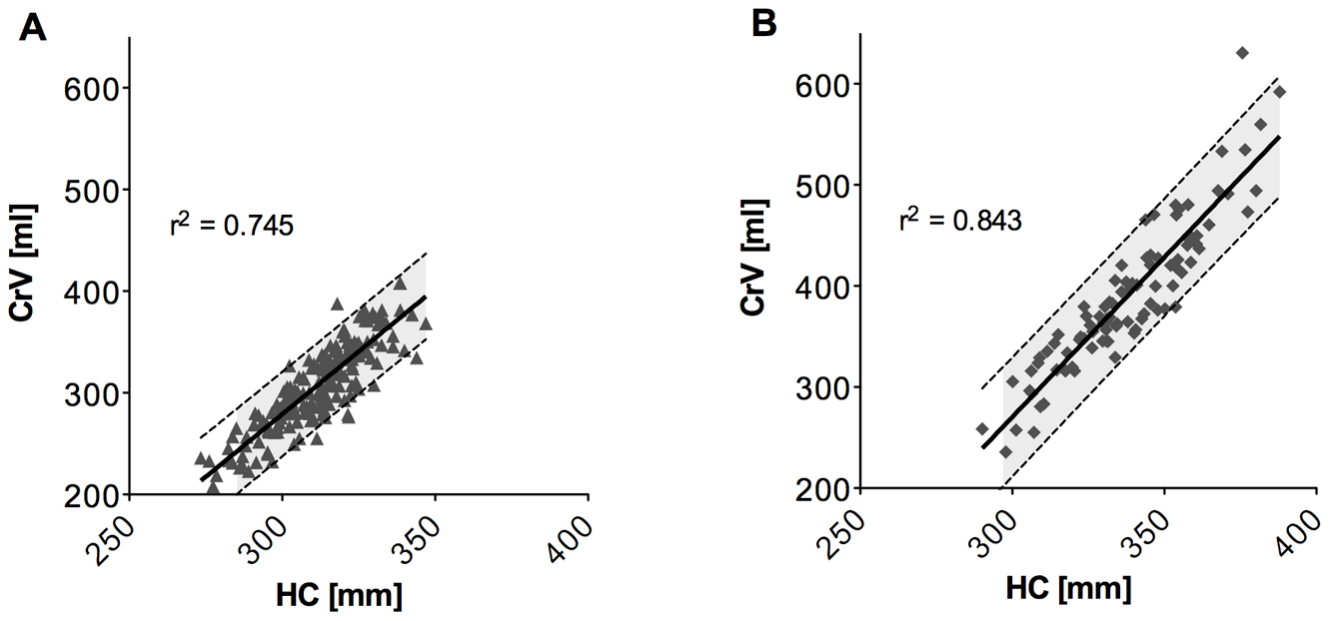
Correlation and prediction intervals of HC and CrV at different Postmenstrual Ages (PMA). CrV plotted against HC for infants A) below 37 weeks PMA and B) greater/equal 37 weeks PMA. Grey continuous line is indicating regression line. Dashed lines and light grey filling define the 95% prediction intervals.

As shown in figure 7 correlation of HC and CrV was worse in infants with a postmenstrual age (PMA) <37 weeks of gestation (r^2^ = 0.745, RSD ±22 ml) compared to older infants (r^2^ = 0.843, RSD ±29 ml).

## Discussion

Head growth is of substantial importance for long-term development of preterm infants. Thus, monitoring of head growth is crucial to optimize clinical care. Whereas currently cranial ultrasound (cUS) and MRI are used to detect and monitor cerebral lesions, sequential manual measurements of head circumference (HC) are used to monitor head growth in infants without cerebral lesions. However, the two-dimensional head circumference does not necessarily reflect the three-dimensional cranial volume (CrV). Laser aided capture of the head shape could be a promising new method, which offers the opportunity to obtain a three-dimensional view on infants' head and thus to monitor its growth in infants without cerebral lesions. We were able to show that reproducibility of HC and CrV are excellent with that method. Although HC and CrV are closely related, a substantial variation of CrV was found in infants of equal HC. Therefore the current practice of using sequential measures of frontal-occipital head circumference to describe head growth in preterm infants could be misleading, especially in infants with head deformities. A three-dimensional approach of longitudinal measures of CrV would be more appropriate than HC to monitor head growth in preterm infants.

In current practice head growth is monitored by manual HC measurements. Previous investigators showed that interobserver differences in manual HC measurements are of a substantial margin in preterm infants. They identified interobserver limits of agreement between −19.9 and 20.3 mm for manual HC measurements in the clinical context [8]. In contrast to this, inter- and intraobserver differences were exiguous for HC in the present study, using a laser shape digitizer (95% of the expected differences not exceeding 2 mm). According to the presented data we conclude that laser aided digital measurements might offer a more reliable determination of HC than manual measurements do.

Our aim in a further step of this study was to compare digital and manual HC measurements in a cohort of preterm and term infants. Overall differences between the two methods were shown to be within a 5% range. Nonetheless, an error of 1.5 cm for an infant with a HC of 30 cm is substantial. These differences might reflect the poor reliability of manual measurements as described in previous studies [8]. Digitally acquired HC was shown to be slightly higher on average. This could be due to the stockinet used for the scans and a higher amount of compressing hair and soft tissue with the cloth tape. However, average discrepancy between the methods was small and since there was a consistent variability without systematic or proportional differences we at least concluded interchangeable use of both methods.

The frontal-occipital HC represents only a two-dimensional view on infants' heads, whereas the noninvasive laser shape digitizers provide three-dimensional surface captures of the head and information on cranial volume (CrV). The present study aimed to verify the predictive value of HC concerning CrV. Though we showed an acceptable correlation of HC and CrV in a cohort of preterm and term infants, CrV varied substantially in infants of equal HC. Since the increase of the total intracranial volume was shown to be 24.05 ml/week from birth to term in preterm infants [23], a residual standard deviation of 24 ml tends to be clinically relevant especially in the group of preterm infants. The correlation was even worse in immature infants (<37 wks. PMA), most likely due to the positional moulding and head deformities in preterm infants that are caused by immature and softer skull structures [14], [15]. Thus, assessment of head growth by sequential measures of HC could be misleading particularly in the group of preterm infants and CrV could be a better predictor for three-dimensional head growth in infants without cerebral lesions.

Cranial US and MRI are well-established tools to investigate anatomical structures of the brain and thus, to detect cerebral lesions. Previous studies using MRI or CT in preterm and term infants showed a correlation of HC and brain volume (BV) [12], [13]. Differences of BV were considerable in infants of similar HC, supporting the need for a 3D approach. However, quantitative MRI and CT scans as methods of assessing BV are cost-intensive and invasive and therefore not applicable as routine clinical measures in neonates. If structural lesions are excluded by cUS or MRI, laser aided surface capture could be a promising method to approximate BV by measuring CrV. The relation of CrV and BV acquired from quantitative MRI scans should be an object of future investigations in infants without cerebral lesions.

The findings of the present study could have an impact on the clinical care in the first weeks of life. Previous investigators assumed that poor growth in preterm infants primarily reflects inadequate nutrient intake [4], [6] and that insufficient early nutrition exerts an adverse influence on long-term developmental outcome [24]. Thus, accurate measures of cranial volume could be a useful help to assess quality of nutritional care. Since data on three-dimensional head growth of preterm infants are limited, appropriate reference values and percentiles for CrV have to be established. Apart from describing cranial volume, laser shape digitizers could be a powerful tool to investigate head deformities in the early postnatal period. Since both, postnatal head growth and head deformities have been shown to predict neurodevelopmental outcome of preterm infants [9]–[11], [16] a routine monitoring of 3D head shape could help to prevent and predict adverse neurodevelopmental outcomes in preterm infants. Therefore, an outcome study relating CrV and subsequently three-dimensional head growth to neurodevelopment of preterm infants is the object of on going investigations.

In summary, the current practice of measuring frontal-occipital HC for describing head growth in preterm infants could be misleading since it does not represent a three-dimensional approach. The 3D laser-scanning device represents a new and promising method to provide reproducible data of cranial volume and head circumference. Since laser scanning does not provide any data on cerebral structures, imaging techniques are still required. However, after excluding cerebral lesions, a 3D approach of monitoring head growth and shape could significantly help to improve neonatal care and long-term outcomes of preterm infants.
